# Hepatitis C virus NS5A protein binds the SH3 domain of the Fyn tyrosine kinase with high affinity: mutagenic analysis of residues within the SH3 domain that contribute to the interaction

**DOI:** 10.1186/1743-422X-5-24

**Published:** 2008-02-11

**Authors:** Holly Shelton, Mark Harris

**Affiliations:** 1 Institute of Molecular and Cellular Biology, Faculty of Biological Sciences and Astbury Centre for Structural Molecular Biology, University of Leeds, Leeds LS2 9JT, UK; 2 Department of Microbiology, School of Biological Sciences, The University of Reading, Whiteknights, RG6 6AJ, Reading, UK

## Abstract

**Background:**

The hepatitis C virus (HCV) non-structural 5A protein (NS5A) contains a highly conserved C-terminal polyproline motif with the consensus sequence Pro-X-X-Pro-X-Arg that is able to interact with the Src-homology 3 (SH3) domains of a variety of cellular proteins.

**Results:**

To understand this interaction in more detail we have expressed two N-terminally truncated forms of NS5A in *E. coli *and examined their interactions with the SH3 domain of the Src-family tyrosine kinase, Fyn. Surface plasmon resonance analysis revealed that NS5A binds to the Fyn SH3 domain with what can be considered a high affinity SH3 domain-ligand interaction (629 nM), and this binding did not require the presence of domain I of NS5A (amino acid residues 32–250). Mutagenic analysis of the Fyn SH3 domain demonstrated the requirement for an acidic cluster at the C-terminus of the RT-Src loop of the SH3 domain, as well as several highly conserved residues previously shown to participate in SH3 domain peptide binding.

**Conclusion:**

We conclude that the NS5A:Fyn SH3 domain interaction occurs via a canonical SH3 domain binding site and the high affinity of the interaction suggests that NS5A would be able to compete with cognate Fyn ligands within the infected cell.

## Background

Hepatitis C virus is an enveloped RNA virus that is estimated to infect 2% of the global population, 123 million individuals [1]. The virus has a positive sense RNA genome of 9.5 kb that comprises a single open reading frame encoding a ~3000 residue polyprotein, flanked by 5' and 3' untranslated regions. The polyprotein is cleaved into 10 individual polypeptides by a combination of host-cell and viral proteases, the N-terminal one-third of the polyprotein produces the four structural proteins (Core, E1, E2 and p7), whereas the C-terminal two-thirds comprises the six non-structural proteins (NS2, NS3, NS4A, NS4B, NS5A and NS5B). Use of a sub-genomic replicon system has demonstrated that five of these (NS3-NS5B) are necessary and sufficient to replicate an RNA molecule containing the 5' and 3' untranslated regions of the viral genome. However, apart from the RNA-dependent RNA polymerase (NS5B), the precise details of the roles of each of the non-structural proteins in the process of RNA replication remain undefined.

**Figure 1:**
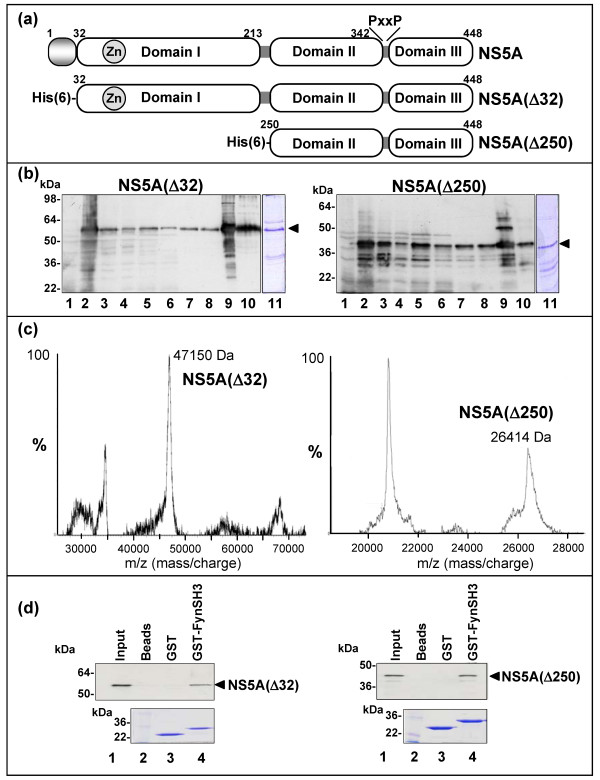
**Expression and purification of N-terminally truncated forms of NS5A**. (a) Schematic of the structure of NS5A and the expressed truncated forms showing the locations of the N-terminal amphipathic helix and the three domains. (b) Anti-NS5A western blot analysis and Coomassie staining of the purification of NS5A(Δ32) (left) and NS5A(Δ250) (right). Lanes: 1; uninduced lysate, 2; induced lysate, 3; lysate clarified by centrifugation and applied to the Ni-NTA column, 4; flow-through, 5–8; washes, 9; 200 mM imidazole elution, 10; gel filtration eluate, 11; Coomassie stain of gel filtration eluate. (c) Slow crystallization mass spectrometry of NS5A(Δ32) (left) and NS5A(Δ250) (right). The peak at ~69 kDa on the left represents the *E. coli *chaperone protein DnaK. The measured molecular masses correspond closely to the predicted values: NS5A(Δ32): 47,222 Da, and NS5A(Δ250): 26,500 Da. (d) Interaction of truncated NS5A forms with the Fyn SH3 domain. Purified NS5A(Δ32) (left) and NS5A(Δ250) (right) were subjected to GST pulldown analysis using GA-beads alone (lanes 2), GST (lanes 3) or GST-FynSH3 (lanes 4). Samples were blotted for NS5A, lanes 1 show 20% of input protein, lanes 2–4 show bound protein eluted by competition with 20 mM reduced glutathione. The lower panel shows a Coomassie stained SDS-PAGE of the purified GST-FynSH3 domain fusion proteins.

NS5A is a 448 amino acid phosphoprotein that interacts with a plethora of cellular proteins and has been reported to have multiple effects on cell physiology, for review see [2]. At the N-terminus of NS5A is a 31 residue amphipathic helix that mediates association of the protein with cytoplasmic membranes [3], this is followed by three domains, separated by short flexible linker regions [4] (Figure 1a). The three-dimensional structure of domain I has been determined and it has been shown to complex with a zinc ion and (at least in the crystal structure) exists as a dimer [5]. The structures of domains II and III remain undetermined but of particular interest is the observation that the flexible linker between these domains contains two motifs with the consensus sequence Pro-X-X-Pro-X-Arg/Lys. We, and others, have shown that these motifs (termed PP2.1 and PP2.2) bind to the Src homology 3 (SH3) domains of a range of cellular proteins. In particular, work has focussed on the PP2.2 motif which is conserved throughout all HCV isolates of all genotypes (unlike the PP2.1 motif which is only conserved in genotype 1 isolates). The PP2.2 motif binds to the SH3 domains of the Src-family kinases Fyn, Lyn, Hck and Lck [6], as well as the adaptor proteins Grb2 [7] and amphiphysin II (also known as BinI) [8,9]. Mutation of the PP2.2 motif also abrogated the ability of NS5A to inhibit activation of the Ras-Erk MAPK pathway [10,11] thus implicating a role for NS5A:SH3 domain interactions in this process. However, the role of the PP2.2 motif in virus replication is controversial; although a mutation of this motif in the context of the genotype 1b sub-genomic replicon had variously either no effect [11], or exhibited a minimal reduction [8,9] in viral RNA replication, it was also reported that a full-length infectious genome containing the same mutation was unable to establish an infection in a chimpanzee [9].

A number of other viral proteins interact with host cell SH3 domains – the best characterised of these is the HIV-1 Nef protein. The interaction between Nef and the SH3 domain of the Src-family kinase, Hck, is reported as one of the strongest interactions between an SH3 domain and its ligand (K_D _= 250 nM) [12], results in activation of the kinase and has been shown to be required for viral pathogenesis *in vivo *[13]. The affinity of a Nef derived PxxPxR-containing peptide for the Hck SH3 domain was much lower than that of the intact protein (K_D _= 91 μM), suggesting that the interaction between Nef and the Hck SH3 domain involved additional intermolecular interactions. We were therefore interested to determine the molecular details of the NS5A:SH3 domain interaction. As an initial approach to this question we used the crystal structure of Nef complexed with a mutated form of the Fyn tyrosine kinase SH3 domain (R96I) [14] as the basis for a molecular modelling study to predict the residues involved in the interaction between the NS5A PP2.2 motif and the Fyn SH3 domain [11]. The results of this study predicted that NS5A would interact with the SH3 domain in a very similar fashion to Nef, and the work presented here was designed to evaluate this prediction using both surface plasmon resonance and mutagenesis of the Fyn SH3 domain. The data confirm the predictions, and furthermore show that NS5A interacts with the Fyn SH3 domain with a similar affinity to that exhibited by the Nef:SH3 domain interaction. We conclude that NS5A binds to SH3 domains with high affinity, and such interactions could occur in the context of an HCV infected cell.

## Results and discussion

We, and others, have previously shown that a conserved C-terminal polyproline motif in NS5A interacts with the SH3 domains of a range of cellular proteins. Although the functional consequences of these interactions remain to be elucidated, recent evidence suggests that this motif may be important for virus replication [9], and thus represents a valid target for antiviral drug development. We therefore performed a detailed biochemical and biophysical analysis of the interaction between NS5A and SH3 domains. To facilitate this analysis we expressed two N-terminally deleted forms of NS5A in *E. coli *– firstly NS5A(Δ32), in which the membrane anchoring amphipathic helix was removed to aid solubility [15]. Secondly, as we had previously shown that the N-terminal 270 residues were dispensable for SH3 domain binding (Andrew Macdonald, PhD thesis, University of Leeds), we expressed NS5A(Δ250) in which both the amphipathic helix and domain I [4] were deleted. Both proteins were expressed with an N-terminal hexahistidine tag to aid purification. Figure 1a shows a schematic of the expressed proteins and figure 1b western blot analysis and Coomassie Blue staining of various stages in the purification process. We established a two stage purification protocol in which NS5A was first purified via the hexahistidine tag by immobilised metal affinity chromatography and further purified by gel filtration (lanes 10). Using this protocol, both forms of NS5A could be purified to approximately 80% purity as judged by Coomassie blue staining (lanes 11). The two forms of NS5A migrated on SDS-PAGE with apparent molecular masses of 55 kDa (Δ32) and 40 kDa (Δ250). To confirm that the expressed proteins were the correct molecular mass they were subjected to slow crystallisation mass spectrometry [16], figure 1c demonstrates that the actual molecular masses were in close agreement with predicted masses. Of note, the apparent molecular masses of each NS5A derived protein species (as indicated by the aberrant migration of the protein on SDS-PAGE) were significantly higher than the actual molecular masses, this is most likely due to the high proline content of NS5A (11% in the intact protein).

We had previously shown that NS5A bound to the SH3 domain of the Fyn tyrosine kinase and that in the context of the intact kinase this interaction led to Fyn activation. As Fyn is expressed in Huh7 cells [6] that are permissive for HCV replication we chose to use the Fyn SH3 domain as a basis for our investigation. We first confirmed that the bacterially expressed NS5A was able to bind a GST-Fyn SH3 domain fusion protein *in vitro *(figure 1d), in agreement with our previous data both forms of NS5A (Δ32 and Δ250) bound equally well. This observation also demonstrated that eucaryotic post-translational modifications were not required for the NS5A-SH3 domain interaction.

**Figure 2:**
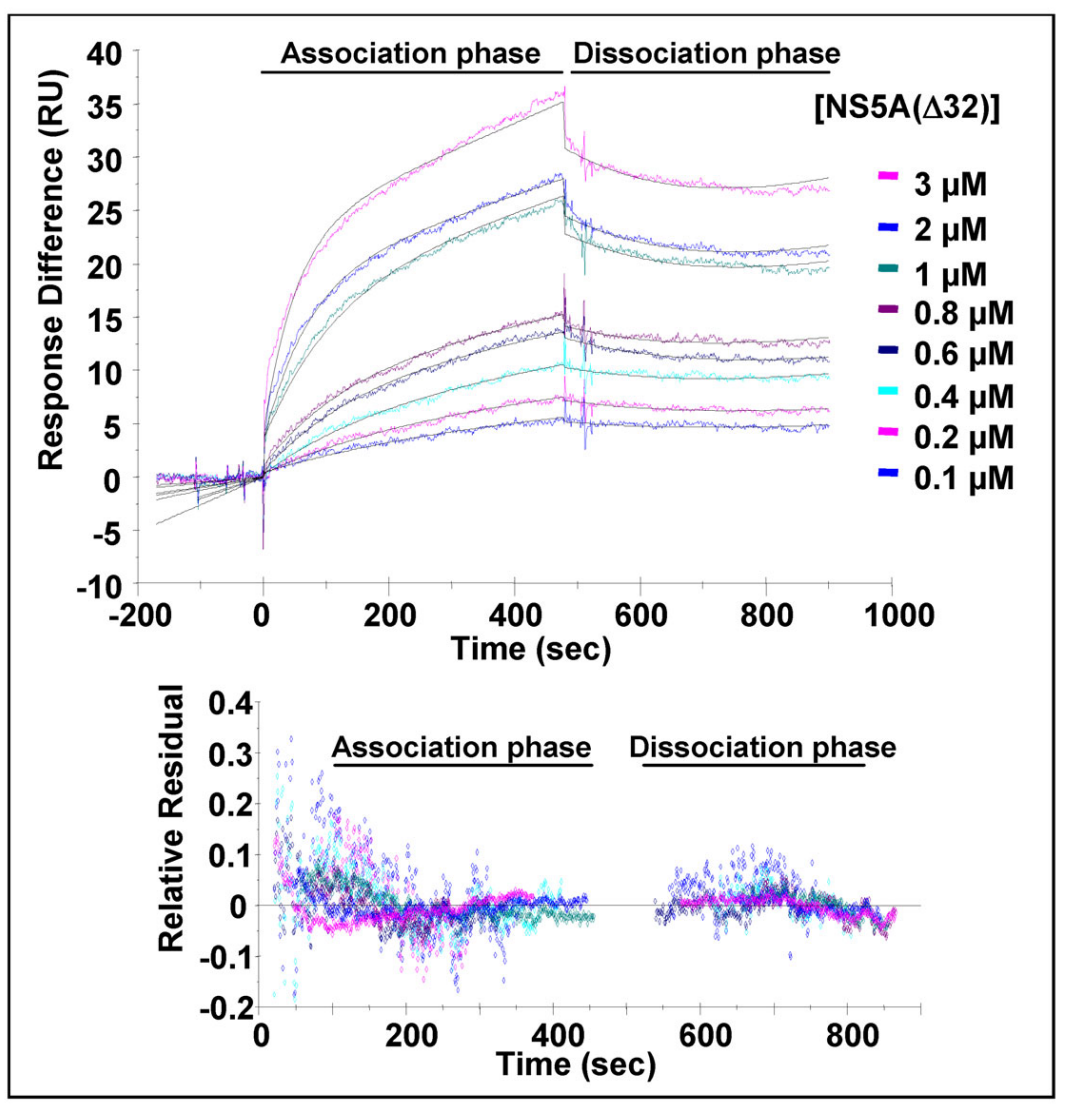
**Surface plasmon resonance analysis of the NS5A(Δ32):Fyn SH3 domain interaction**. SPR analysis was performed as described for NS5A(Δ32) and GST-FynSH3. The indicated concentrations of NS5A were flowed over a sensor cell on to which GST-FynSH3 was captured via an anti-GST antibody. The generated fit is shown as black lines in the top panels, the residuals plotted in the lower panels indicate the relative response difference between the generated fit and the raw data – demonstrating the closeness of fit.

**Figure 3:**
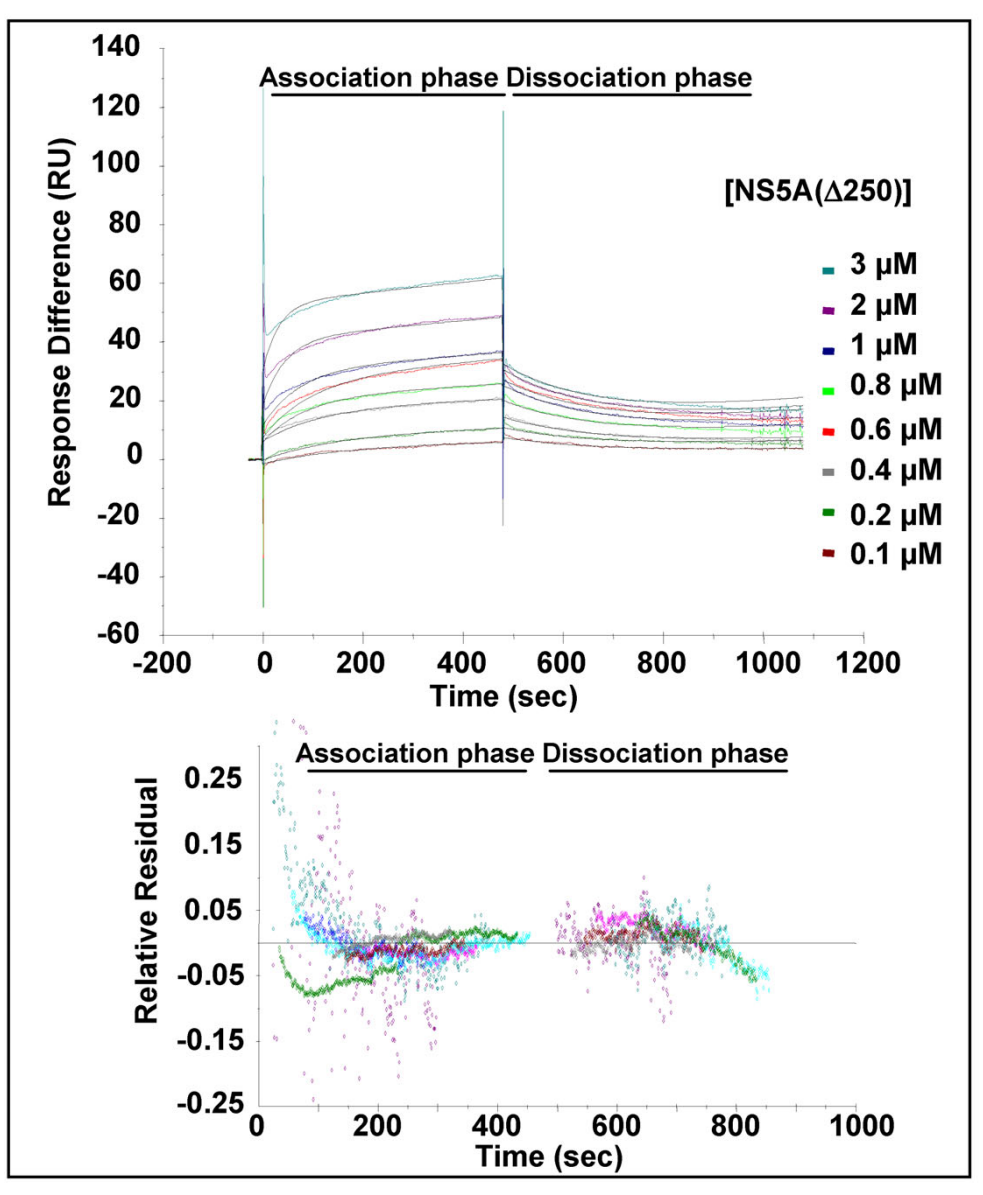
**Surface plasmon resonance analysis of the NS5A(Δ250):Fyn SH3 domain interaction**. SPR analysis was performed as described for NS5A(Δ250) and GST-FynSH3. The indicated concentrations of NS5A were flowed over a sensor cell on to which GST-FynSH3 was captured via an anti-GST antibody. The generated fit is shown as black lines in the top panels, the residuals plotted in the lower panels indicate the relative response difference between the generated fit and the raw data – demonstrating the closeness of fit.

To obtain quantitative data about the affinity of the interaction between NS5A and the Fyn SH3 domain we utilised surface plasmon resonance. We first confirmed that we could detect no binding of either form of NS5A to either sensor chips loaded with anti-GST antibody alone, or anti-GST antibody with the addition of GST (data not shown). Purified NS5A was then flowed over sensor chips on to which were immobilised GST-FynSH3, as shown in figures 2 and 3 this analysis revealed that both forms of NS5A bound the Fyn SH3 domain with high affinity – for NS5A(Δ32) the K_D _was calculated at 629 nM, for NS5A(Δ250) 556 nM. The individual values of k_d _and k_a _used to calculate the K_D _values are presented in Table 1. This data confirmed that domain I of NS5A was not involved in interactions with the SH3 domain, however we cannot rule out the possibility that domain I might mediate interactions with the intact kinase. Interestingly, the affinity of NS5A for Fyn SH3 was similar to that determined for the HIV-1 Nef protein binding to either Hck SH3 or a mutant Fyn SH3 (R96I) (250 nM). In the case of Nef it has been shown that the high affinity of the interaction was mediated not only by interactions between residues in the SH3 domain and the polyproline motif, but also by other interactions, for example involving the RT loop of the SH3 domain.

**Table 1:** Kinetic parameters for the NS5A:Fyn SH3 domain interaction.

**Protein**	**k_a _(M^-1^s^-1^)**	**k_d _(s^-1^)**	**K_D _(M^-1^)**
NS5A(Δ32)	4.93 × 10^3^	3.08 × 10^-3^	6.29 × 10^-7^
(± SD) n = 3	(± 0.66 × 10^3^)	(± 0.16 × 10^-3^)	(0.59 × 10^-7^)
NS5A(Δ250)	9.05 × 10^3^	4.94 × 10^-3^	5.56 × 10^-7^
(± SD) n = 3	(± 1.62 × 10^3^)	(± 0.11 × 10^-3^)	(0.59 × 10^-7^)

**Figure 4:**
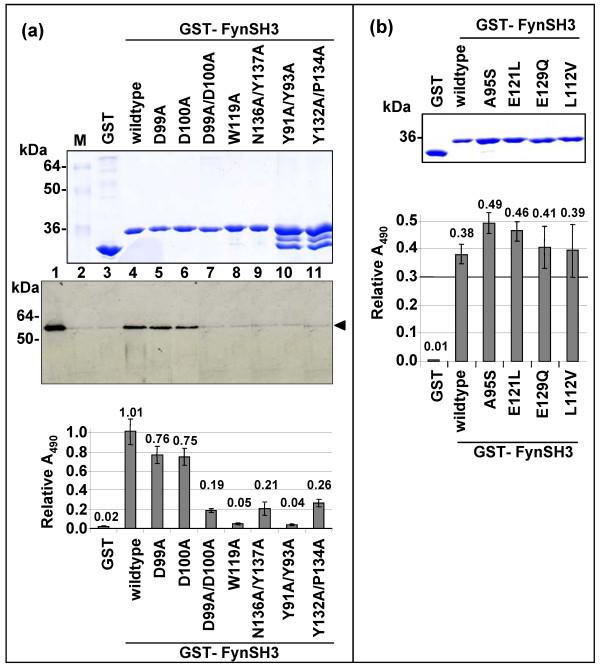
**Mutational analysis of the NS5A:FynSH3 domain interaction**. The indicated mutations were generated in the Fyn SH3 domain – the numbering refers to the residues in the full length Fyn protein. For reference the Fyn SH3 domain commences at residue 84 [17]. (a) Top panel – Coomassie stained SDS-PAGE of the purified GST-FynSH3 domain fusion proteins. Lower panel – GST pulldown analysis. Purified NS5A(Δ32) was subjected to GST pulldown analysis using GA-beads alone (lane 2), GST (lane 3) or GST-FynSH3 or mutants thereof (lanes 4–11 – corresponding to the GST-FnSH3 mutants in the lanes above). Samples were blotted for NS5A, lane 1 shows 20% of input protein, lanes 2–11 show bound protein eluted by competition with 20 mM reduced glutathione. ELISA analysis. GST-FynSH3 domain fusion proteins were immobilised on microplates and incubated with purified NS5A(Δ32), prior to detection of bound NS5A with sheep anti-NS5A serum. Absorbance values were normalised to the amount of GST fusion proteins loaded (measured by direct ELISA with an anti-GST antibody). The mean value determined over 3 independent experiments is indicated on the graph. (b) Coomassie stained SDS-PAGE of the purified GST-FynSH3 domain fusion proteins and ELISA analysis as in (a).

Based on the crystal structure of the Nef-Fyn(R96I)SH3 domain complex we had previously predicted that both Nef and NS5A would make the same intermolecular contacts with the SH3 domain [11]. Four residues in the SH3 domain, namely D100, W119, N136 and Y137, were predicted to make major contributions to the binding energy of the NS5A-SH3 domain interaction. To test this hypothesis we constructed the corresponding site-directed mutants of the Fyn SH3 domain in the context of a GST-FynSH3 domain fusion, replacing the indicated residues with alanine. These were purified and tested for binding to NS5A both by GST-pulldown and ELISA (figure 4a). As expected from the molecular modelling analysis, both W119A and the double mutant N136A/Y137A abrogated binding, however D100A had no effect. This was unexpected as our previous study had predicted that D100 would form a salt bridge with residue R356 in NS5A, however, as D100 was preceded by an additional acidic residue (D99) it was plausible that R356 could form a salt bridge with either acidic residue. To test this hypothesis we constructed two further mutants, D99A and a double substitution D99A/D100A. Gratifyingly, this analysis revealed that D99A had no effect, however the double mutant dramatically reduced binding (figure 4a) – thus it appears that there is some flexibility in the interaction as R356 within NS5A can form a salt bridge with either of two adjacent acidic residues in the SH3 domain. Interestingly the side chain of residue D100 has also been shown to make an intramolecular hydrogen bond with R96 within the RT-Src loop of the SH3 domain – it was possible that the effects of the double mutant were due to the inability to form this bond – thus perturbing the correct folding of the SH3 domain. However, a comparison of the circular dichroism spectra of the four GST-FynSH3 fusions (wildtype, D99A, D100A and D99A/D100A) revealed no significant differences in the overall fold of the proteins (data not shown), suggesting that this is not the case. In addition we generated two mutants of residues predicted to make a minor contribution to the interaction – Y91A/Y93A and Y132A/P134A. Interestingly these mutations had a significant impact on the binding to NS5A, although as can be seen in figure 4a these two mutant SH3 domains were somewhat unstable and exhibited degradation to GST.

Interestingly, of the nine residues predicted to contribute to the binding energy of the interaction, eight were conserved between Fyn and c-Src (the only difference being D99 in the RT-loop – altered to T99 in c-Src). Despite this both we [6] and others [7] had previously observed that NS5A was unable to bind to the SH3 domain of Src so to determine if other residues in the SH3 domain might make a contribution to the interaction we proceeded to make a second set of mutations. In order to avoid any potential structural effects we chose to mutate four residues in Fyn to their corresponding c-Src residues. Three of these were non-conservative changes: A95S, E121L and E129Q, the fourth was a conservative change, L112V. We therefore made the corresponding mutations within the Fyn SH3 domain, expressed them as GST-fusion proteins and tested these mutant proteins for binding to NS5A by ELISA (figure 4b). None of these mutations had any significant effect on the binding to NS5A.

Our data are consistent with the notion that the PP2.2 polyproline motif of NS5A is a promiscuous and high affinity SH3 ligand, able to mediate binding of NS5A to a wide range of SH3 domains. The binding of NS5A to the Fyn SH3 domain is remarkably resistant to single or multiple amino acid substitutions, apart from the relatively well conserved residues – tyrosines 91 and 93 and tryptophan 119 – mutation of which reduced binding to background levels. These three residues have previously been shown to play critical roles in peptide binding and are highly conserved [17], thus the dramatic effect on NS5A binding is in line with expectations. The affinity of NS5A for the Fyn SH3 domain is higher than the corresponding values for most cellular SH3 domains interacting with their cognate ligands – generally such interactions have calculated K_D _between 1–50 μM [17], although some have been reported to have much higher affinities, eg the amphiphysin:dynamin I interaction was measured at 190 nM [18]. The comparison of binding affinities suggests that NS5A would be able to effectively compete with cellular ligands for binding to SH3 domains. In this context it will be of great interest to determine which SH3 domain containing proteins interact with NS5A in cells infected with HCV, such experiments are ongoing in our laboratory. It is interesting to note that a recent study [19] pointed to a key role for Fyn in HCV RNA replication. However, in apparent contrast to our previous data demonstrating activation of Fyn by NS5A, this study showed that Fyn activation via phosphorylation mediated by the upstream kinase, Csk, resulted in inhibition of replicon replication.

## Methods

### Protein expression and purification

Coding sequences for the two N-terminally truncated forms of NS5A were amplified by PCR using the J4 genotype 1b clone of HCV [20] as template and cloned into pET14b. Primer sequences are available upon request. Protein expression was carried out in *E. coli *BL21 pLysS (DE3). Briefly, a single colony was grown in LB containing 100 μg/ml ampicillin, 50 μg/ml chloramphenicol and 1% (w/v) glucose at 37°C until OD_600 _= 0.6. The culture was chilled at 4°C for 30 minutes prior to induction with 0.2 mM IPTG at 27°C for 5 hours. Bacterial pellets were resuspended in buffer A (20 mM disodium orthophosphate, pH 7.5, 0.5 M NaCl, 5 mM MgCl_2_), containing 1 mg/ml lysozyme, 2 μg/ml DNase, 1 μg/ml RNase, 0.5% Triton X-100 and EDTA-free complete protease inhibitor cocktail (Roche), sonicated, clarified by centrifugation (16,000 × g at 4°C for 1 hour) and applied to a Ni^2+^-charged NTA-sepharose column. The column was washed extensively in buffer A containing 20 mM imidazole and eluted in the same buffer containing 300 mM imidazole and 0.1% Triton X-100. NS5A-containing fractions were pooled, dialysed overnight against HBS buffer, clarified by centrifugation and injected onto an Amersham XK 16/70 size exclusion column packed with Superdex75 (Amersham Biosciences). Fractions containing intact protein were again pooled and stored at -80°C until use. GST-SH3 domain fusion proteins were expressed and purified as previously described [6].

### GST pulldown assay

GST-SH3 domain fusion proteins were bound to glutathione agarose (GA)-beads at 4°C for 1 h. 5 μg of purified NS5A protein was applied to the beads and incubated at 4°C on a blood mixer for 3 hours. The beads were washed twice in GLB (10 mM PIPES-NaOH pH 7.2, 120 mM KCl, 30 mM NaCl, 5 mM MgCl_2_, 1% (v/v) Triton X-100, 10% (v/v) glycerol) supplemented with 0.5 M KCl, and three times in GLB only. Bound proteins were eluted from the GA-beads by incubating in the presence of 20 mM reduced glutathione in GLB. Samples were analysed by western blot and Coomassie stained SDS-PAGE to confirm equal GST-SH3 domain fusion protein loading onto the GA-beads.

### ELISA assay

1 μg/well of either GST or the appropriate GST-SH3 domain fusion proteins in 50 μl of PBS was coated on Greiner bio-One PS 96-well microplates overnight at 4°C. Wells were rinsed with PBS/0.1% (v/v) Tween-20 (PBS-T) and blocked in PBS-T containing 5% (w/v) dried semi-skimmed milk powder (PBS-TM) for 2 hours at room temperature. After washing in PBS-T, 0.5 μg/well of purified NS5A was added in 50 μl of PBS-T and incubated for 2 hours at 4°C. Bound NS5A was detected with a sheep polyclonal anti-NS5A sera (1:5000 dilution) in PBS-TM followed by donkey anti-sheep-HRP (Sigma). The ELISA was developed using OPD, the reaction was stopped with 0.5 M sulphuric acid. The ELISA plate was read at 490 nm (referenced at 630 nm) using an MRX plate reader (Dynex). All samples were run in triplicate and an average taken in each case. To control for differential binding of the GST-SH3 domains to the 96-well microplate, a GST loading ELISA was utilised. In this case the primary antibody was a mouse anti-GST mAb (Serotec), and the secondary was a goat anti-mouse HRP conjugate (Sigma). The amount of relative binding for each GST-SH3 fusion protein was analysed and the signal from the NS5A ELISA normalised appropriately.

### Surface Plasmon Resonance

Experiments were carried out on a Biacore 3000 in HBS buffer (10 mM Hepes-NaOH pH 7.4, 150 mM NaCl, 0.005% Tween-20) at 25°C. An anti-GST antibody (Biacore) was amine coupled to a CM5 sensor chip which allowed the capture and immobilisation of 2 μg of purified GST-SH3 domains in a uniform orientation to generate a binding surface. A reference surface containing antibody only was also prepared. Eight concentrations of purified NS5A(Δ32) or NS5A(Δ250) (3 μM – 100 nM), were injected across the binding or reference sensor chip surface at 30 μl/minute for 8 minutes followed by 8 minutes dissociation, in triplicate. A corrected binding profile was then generated by subtraction of the reference signal from the binding signal for each concentration. A 1:1 Langmuir binding curve was applied to the concentration series of each protein and the association (k_a_), dissociation (k_d_) rates and affinity constant (K_D_) were calculated.

## Abbreviations

HCV: hepatitis C virus; NS5A: non-structural 5A protein; SH3: Src homology 3.

## Competing interests

The author(s) declare that they have no competing interests.

## Authors' contributions

MH conceived and supervised the study. HS performed the experiments. MH wrote the manuscript. Both authors read and approved the final manuscript.
